# Soluble CD93 in allergic asthma

**DOI:** 10.1038/s41598-019-57176-2

**Published:** 2020-01-15

**Authors:** Hye Jung Park, Eun-Yi Oh, Hee-Jae Han, Kyung Hee Park, Kyoung-Yong Jeong, Jung-Won Park, Jae-Hyun Lee

**Affiliations:** 1 Department of Internal Medicine, Gangnam Severance Hospital, Seoul, Republic of Korea; 2 Institute of Allergy, Yonsei University College of Medicine, Seoul, Republic of Korea; 3 Division of Allergy and Immunology, Department of Internal Medicine, Yonsei University College of Medicine, Seoul, Republic of Korea

**Keywords:** Diagnostic markers, Predictive markers

## Abstract

CD93 has been shown critical roles in inflammatory and immune diseases. However, in allergic asthma, the potential roles of soluble CD93 (sCD93) have not been well studied. We conducted house dust mite (HDM) stimulation with Der p 1 in BEAS-2B and U937 cells, followed by treatment with dexamethasone or small interfering RNA against CD93. A HDM-induced murine allergic asthma model was also established. We estimated the power of sCD93 to predict allergic asthma in a retrospective post-hoc analysis containing 96 human samples. HDM-stimulated BEAS-2B cells showed increased mRNA expression levels of IL-6, IL-8, IL-33, TSLP, and CD93. The CD93 level in culture supernatants steadily increased for 24 h after allergen stimulation, which was significantly suppressed by both dexamethasone and CD93 silencing. CD93 silencing increased IL-6 and TSLP, but not IL-33 levels in culture supernatants. HDM-induced asthma mice showed significant airway hyperresponsiveness and inflammation with Th2 cytokine activation, along with decreased CD93 expression in bronchial epithelial cells and lung homogenates but increased serum CD93 levels. The sCD93 level in asthma patients was significantly higher than that in healthy controls and could predict asthma diagnosis with moderate sensitivity (71.4%) and specificity (82.4%) (AUC = 0.787, *P* < 0.001). The level of sCD93 which has potential role to predict asthma significantly increased after HDM stimulation via IL-6 and TSLP *in vitro* and *in vivo*.

## Introduction

Asthma is a chronic inflammatory airway disease that is typically diagnosed based on a pulmonary function test with a bronchodilator and a bronchial provocation test^1^. However, these tests require the effort and cooperation of the patient, which is often impossible owing to old age, deafness, misunderstanding, severe dyspnea, paroxysmal cough, and risk of an asthma attack^2^. Therefore, there is a clinical need to identify a serum biomarker that can act as a non-invasive method of diagnosis for asthma patients; although several biomarkers have been suggested, they are not widely used in clinical settings owing to limited evidence^3,4^.

CD93 (C1qRp) is a transmembrane glycoprotein that is expressed on various cell types, including endothelial cells, epithelial cells, stem cells, platelets, and leukocytes^5,6^. CD93 can be shed in a soluble form (sCD93) in response to inflammatory mediators, can be easily measured, and is considered to be associated with various inflammatory and immune associated diseases, including asthma^7,8^. In addition, CD93 has angiogenic and growth-stimulating effects^9–11^. Raedler *et al*.^12^ showed that CD93 gene expression was significantly higher in non-allergic asthma patients than healthy controls, and Sigari *et al*.^13^ demonstrated that the serum CD93 level increased under asthma exacerbation and decreased following proper treatment. We also previously reported the potential of sCD93 as a novel biomarker for asthma using an ovalbumin-induced asthma murine model and human patients^14,15^. However, the changes of sCD93 after allergic stimulation have not been assessed *in vitro*, and the potential of sCD93 as a biomarker to help in asthma diagnosis remains unknown.

Toward this end, the aim of this study was to assess the roles of sCD93 in allergic inflammation using *in vitro* and in *vivo* analysis with cell and murine models induced using house dust mite (HDM) extract. We further assessed the usability of sCD93 for asthma diagnosis by a retrospective post-hoc analysis of the CD93 levels^14^. Since the serum sCD93 level can be easily obtained and interpreted objectively, clarifying the potential role of serum CD93 in asthma patients would provide a valuable tool for clinical diagnosis and management.

## Methods

### Cell culture

Two cell lines, BEAS-2B derived from human bronchial epithelial cells (American Type Culture Collection, Rockville, MD, USA) and U937 derived from human histiocytic lymphoma cells (Korean cell line bank, Seoul, Korea) were cultured according to the manufacturer’s instructions until reaching 90% confluence in 6 wells in the incubator. BEAS-2B cells were cultured in BEGM^®^ (Lonza, Clonetics^®^, Basel, Switzerland) and U937 cells were cultured in RPMI (Gibco Laboratories, Grand Island, NY, USA) with 10% fetal bovine serum (Hyclone Laboratories, Logan, UT, USA). The experiment was performed triplicate. The U937 cells were used because they are easily stimulated by HDM^16^.

### Allergen stimulation

The cells were treated with HDM extract of group I major allergen (Der p 1) at various concentrations (0, 0.01, 0.1, 1.0 μg/mL) and times (0, 1, 2, 4, 24 h). HDM (*Dermatophagoides pteronyssinus*, Institute of Allergy, Yonsei University, Seoul, Korea) were cultured in an insect rearing facility, and extracted as previously described^17,18^. The HDM were then pulverized, defatted, and extracted at 4 °C overnight. The extracts of HDM were centrifuged, and the supernatants were filtered, and finally used for cell stimulation.

### CD93 small interfering RNA (siRNA) and dexamethasone treatment

The cells were transiently transfected with siRNA targeting CD93 (sequence: GUA GAU AAU GCC CUU CUA U; Bioneer, Daejeon, Korea) to knock down CD93 expression. In brief, the 90% confluent cells were seeded and diluted with Lipofectamine^®^2000 (Invitrogen, CA, USA) reagent in Opi-MEN^®^ medium (Invitrogen, CA, USA), supplemented with DNA and DNA-reagent complex according to the manufacturer instructions. In addition, the cells were treated with 1 μM of dexamethasone (9α-fluoro-16α-methylprednisolone; Sigma−Aldrich, St. Louis, MO, USA) to reduce allergic inflammation based on the previous study^19^.

### Polymerase chain reaction (PCR) and cytokine analysis

The mRNA expression of the cytokines interleukin (IL)-6, IL-8, IL-33, thymic stromal lymphopoietin (TSLP), and CD93 was assessed using a real-time PCR system (StepOnePlus, Applied Biosystems, Foster City, CA, USA) with amplification from synthesized cDNAs. The primers were as follows: GAPDH forward, 5′-AGGGCTGCTTTTAACTCTGGT-3′; GAPDH reverse, 5′-CCCCACTTGATTTTGGAGGGA-3′; IL-6 forward, 5′-AATTCGGTACATCCTCGACGG-3′; IL-6 reverse, 5′-GGTTGTTTTCTGCCAGTGCC-3′; IL-8 forward, 5′-GACCACACTGCGCCAACAC-3′; IL-8 reverse, 5′-CTTCTCCACAACCCTCTGCAC-3′; IL-33 forward, 5′-GTGACGGTGTTGATGGTAAGAT-3′, IL-33 reverse, 5′-AGCTCCACAGAGTGTTCCTTG-3′; TSLP forward, 5′-ATGTTCGCCATGAAAACTAAGGC-3′; TSLP reverse, 5′-GCGACGCCACAATCCTTGTA-3′; CD93 forward, 5′-GCTGTTGCTCTTATCTGCAAGGTG-3′; CD93 reverse, 5′-AGCAAGCCTTTGCAGGGATCTA-3′. The concentrations of IL-6, IL-8, IL-33, TSLP, and CD93 in culture supernatants were assessed by enzyme-linked immunosorbent assays (IL-6, IL-8, IL-33, TSLP kits by R&D Systems, San Diego, CA, USA; CD93 kit by Affymetrix eBioscience, Vienna, Austria) according to the manufacturer instructions. The detection range for CD93 was 3.3–400 ng/mL. All samples were assessed in duplicate.

### Establishment of the HDM-induced asthma murine model

**Figure 1:**
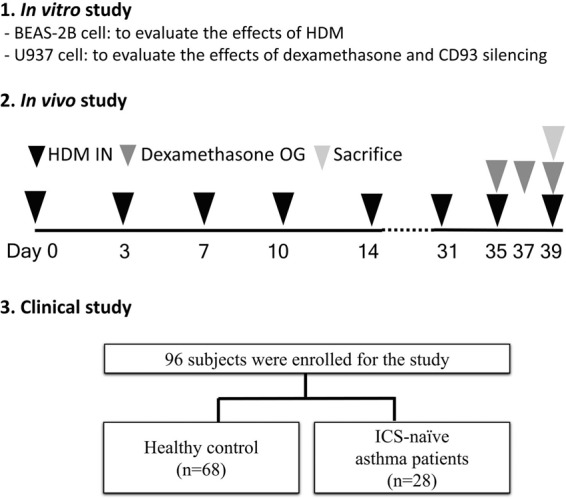
Overview of the study design. HDM: house dust mite, IN: intranasal inoculation, OG: oral gavage, ICS: inhaled corticosteroid.

We already generated OVA-induced asthma model, and confirmed significant roles of sCD93 in OVA-induced asthma model^15^. However, in this study, We generated an HDM-induced asthma model using female BALB/c mice (5 weeks old; Orient, Daejeon, Korea) to match the design to that of cell experiment. The mice were maintained in a conventional animal facility under pathogen-free conditions, and then intranasally administered 30 μg of HDM crude extract (1 mg/mL) two times per week for 6 weeks. The control group was treated in the same manner, but with the same volume of saline. On days 35, 37, and 39, dexamethasone (3 mg/kg) or vehicle was administered by gavage to reverse the allergic inflammation induced by HDM inoculation. All mice were sacrificed after the last treatment on day 39. The number of mice in each group was 5. All experimental procedures of mice studies were approved by the Institutional Animal Care and Use Committee, Animal Research Ethics Board of Yonsei University (Seoul, Korea) (IACUC approval number, 2015–0314) and were performed in accordance with the Committee’s guidelines and regulations for animal care (Fig. 1).

After treatment, the lungs were resected and homogenized using a tissue homogenizer (Biospec Products, Bartlesville, OK, USA) in lysis buffer and protease inhibitor solution (Sigma-Aldrich, St Louis, MO, USA) according to a previous study^15,20^. IL-4, IL-13, IL-5, IL-33, TSLP, IL-6, and CD93 levels in the lung homogenates and the CD93 level in serum were assessed by enzyme-linked immunosorbent assays as described above.

The histopathologic studies were conducted according to a previous study^15,20^. The lung sections were cut and stained with H&E, periodic acid–Schiff (PAS), and Masson’s trichrome (MT). For immunohistochemistry, the slides were deparaffinized and incubated with anti-CD93 antibody (ab198854, Abcam, Cambridge, MA, USA). After washing in phosphate-buffered saline, the slides were treated with goat anti-rabbit IgG H&L (ab150077, Abcam) and incubated. The slides were counterstained with hematoxylin and mounted using mounting medium.

### CD93 levels in asthma patients

We conducted post-hoc analysis using data obtained from our previous study approved by institutional review board of Yonsei university college of medicine (approval number, 4-2013-0397)^14^. Informed consent was obtained from all the subjects. All methods were carried out in accordance with relevant guidelines and regulations. According to the original study, subjects who were in exacerbated status of underlying allergic disease including asthma, in immunotherapy, have activated status of another inflammatory disease, and have severe cutaneous adverse drug reactions were excluded^14^. We selected and enrolled 96 ICS-naïve subjects who were admitted to the Allergy Asthma Center of the Severance Hospital in Seoul, Korea from May 2014 to September 2015. Asthma was diagnosed by an allergy specialist based on clinical guidelines using a bronchodilator test and/or bronchial provocation test^21^. Twenty-eight subjects were finally diagnosed as having asthma, and the others served as the control group (Fig. 1). The serum CD93 level was assessed by enzyme-linked immunosorbent assays as described above.

### Statistical analysis

The two-sample *t*-test (independent sample *t*-test) and analysis of variance followed by a post-hoc Bonferroni test were conducted to compare continuous variables among the subgroups. After adjustment for age and sex, receiver-operator characteristic (ROC) analysis was conducted to obtain the area under the curve (AUC), sensitivity, and specificity of the serum CD93 level to diagnose asthma. A *P* value less than 0.05 was considered statistically significant.

## Results

### Changes of mRNA expression after HDM stimulation *in vitro*

**Figure 2:**
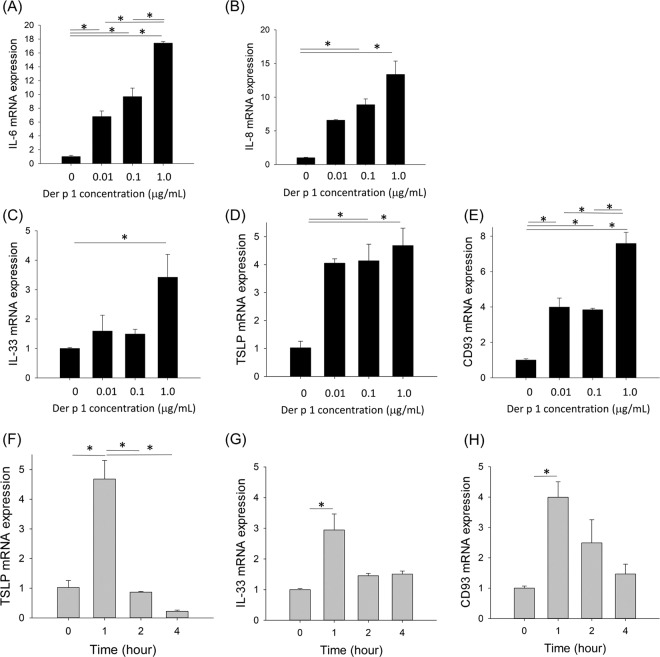
Changes of IL-6 (**A**), IL-8 (**B**), IL-33 (**C**), TSLP (**D**), and CD93 (**E**) mRNA expression levels according to the treated Der p 1 concentration in BEAS-2B cells, and changes of TSLP (**F**), IL-33 (**G**), and CD93 (**H**) mRNA expression levels according to the time of treatment with 0.1 μg of Der p 1 in BEAS-2B cells. *P < 0.05.

HDM was treated with various concentrations of Der p 1 (0, 0.01, 0.1, 1.0 μg/mL) for 1 hour in BEAS-2B cell. The mRNA expression of IL-6, IL-8, IL-33, TSLP, and CD93 was steadily increased according to the increase of Der p 1 concentration. (Fig. 2A–E). We further treated the BEAS-2B cells with 0.1 μg/mL of Der p 1 for various times to induce a significant increase in the mRNA expression levels. The mRNA expression levels of TSLP, IL-33, and CD93 significantly increased after 1 h and then sharply decreased to the baseline level at 2 and 4 h after HDM treatment (Fig. 2F–H).

### Changes of cytokine levels after dexamethasone or CD93 siRNA treatment

**Figure 3:**
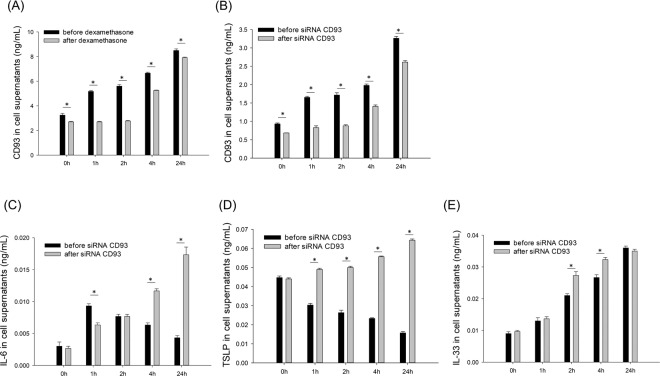
Changes of CD93 levels in culture supernatants before and after dexamethasone treatment. (**A**) Changes of CD93 levels in culture supernatants before and after siRNA CD93 treatment. (**B**) Changes of IL-6 (**C**), TSLP (**D**), and CD93 (**E**) levels in culture supernatants before and after siRNA CD93 treatment. *P < 0.05.

After treatment with 0.1 μg/mL of Der p 1, the CD93 level in the culture supernatants steadily increased with time and then significantly decreased with dexamethasone treatment (Fig. 3A). CD93 silencing induced a significantly decreased level of CD93 in culture supernatants to the same degree observed with dexamethasone (Fig. 3B).

The level of IL-6 in the culture supernatants significantly increased after 1 h and then decreased at 2, 4, and 24 h after HDM stimulation. However, treatment with CD93 siRNA led to a steady increase in the IL-6 level after 24 h (Fig. 3C). The level of TSLP in culture supernatants significantly decreased with time; however, siRNA treatment increased the TSLP level over time (Fig. 3D). The level of IL-33 steadily increased with time, and siRNA treatment did not significantly affect this trend (Fig. 3E).

### Successful establishment of the HDM-induced asthma mouse model

**Figure 4:**
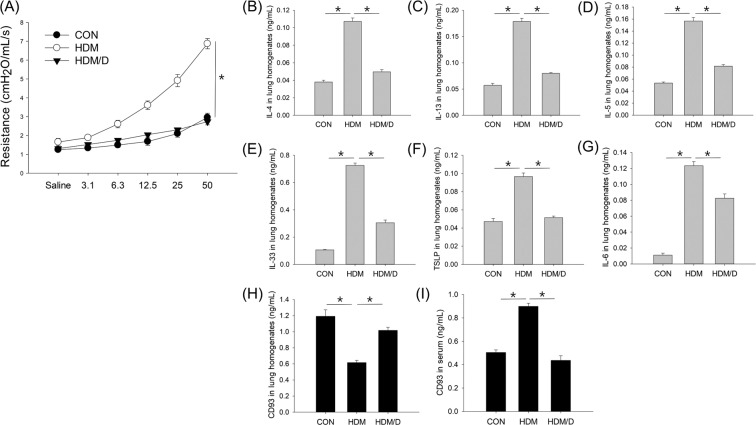
Airway hyperresponsiveness (**A**); IL-4 (**B**), IL-13 (**C**), IL-5 (**D**), IL-33 (**E**), TSLP (**F**), IL-6 (**G**), and CD93 (**H**) levels in lung homogenates; and the CD93 level in serum (**I**) among groups in an HDM-induced asthma mouse model. *P < 0.05.

HDM led to significant airway hyperresponsiveness in the mice, which was fully restored by dexamethasone treatment (Fig. 4A). IL-4, IL-13, IL-5, IL-33, TSLP, and IL-6 levels in the lung homogenates were significantly increased in the HDM group than in the control group, while dexamethasone significantly reversed this cytokine induction (Fig. 4B–G). However, the CD93 level in the lung homogenates was significantly decreased in the HDM group compared to that in the control group. The dexamethasone-treated group showed a significantly higher level of CD93 in the lung homogenates than the HDM group (Fig. 4H). By contrast, the CD93 level in the serum was significantly increased in the HDM group compared to that in the control group; this was fully recovered in the dexamethasone-treated group (Fig. 4I).

### Histology and immunohistochemistry of CD93 in the HDM-induced asthma mouse model

**Figure 5:**
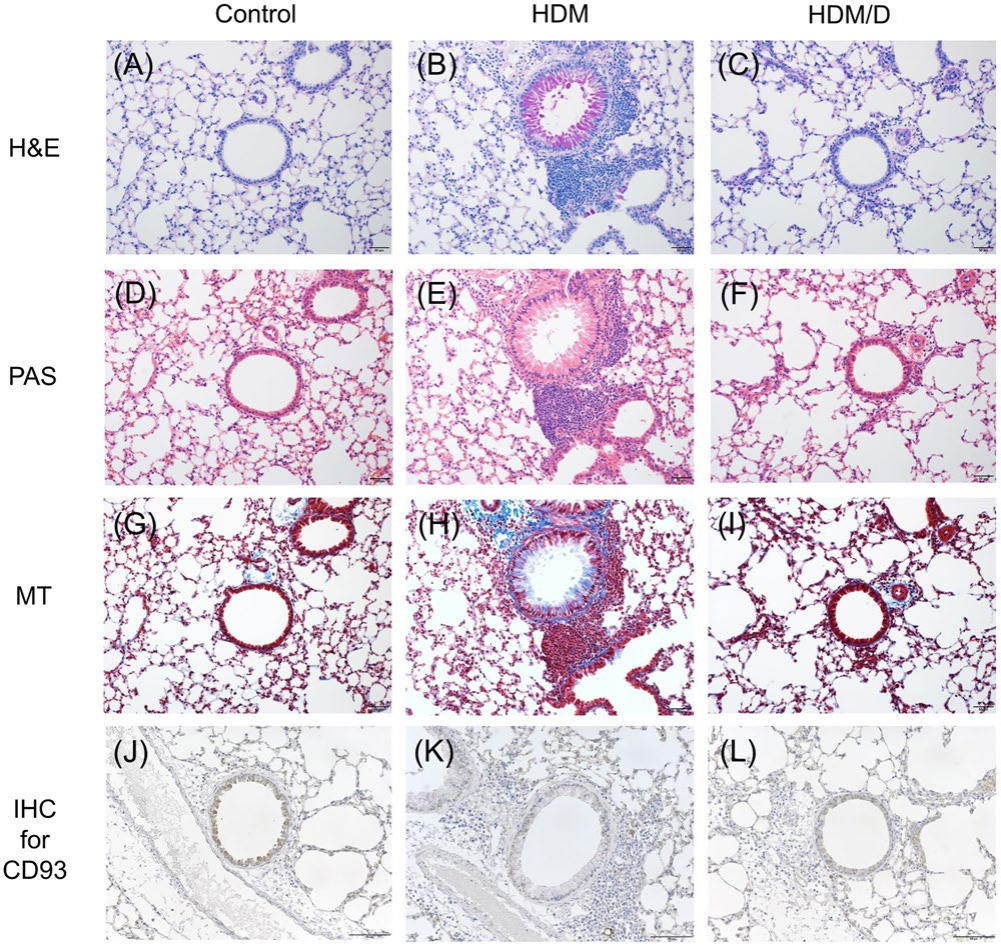
Pathologic analysis assessed by H&E (**A**–**C**), periodic acid-Schiff (**D**–**F**), Masson trichrome (**G**–**I**), and anti-CD93 antibody staining for immunohistochemistry (**J**–**L**) among groups in an HDM-induced asthma mouse model.

Histological findings showed abundant infiltration of inflammatory cells (Fig. 5A–C), extensive proliferation of goblet cells (Fig. 5D–F), and extensive fibrosis (Fig. 5G–I) induced by HDM compared to the control group, which were all improved by dexamethasone. Immunohistochemical staining revealed decreased CD93 expression in the bronchial epithelia of the HDM group compared to control group. Although dexamethasone restored the CD93 level to some extent, the overall effect was weak (Fig. 5J–L).

### Predictive power of the serum sCD93 level for asthma diagnosis

**Figure 6:**
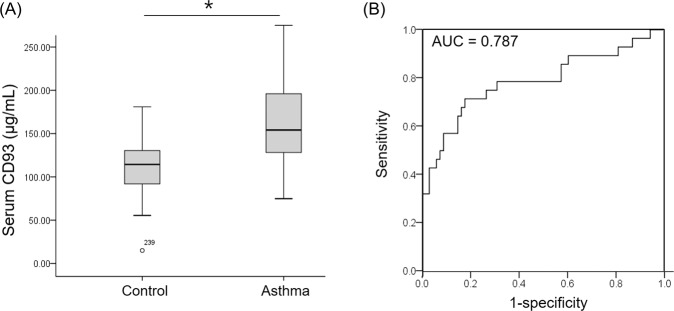
Significant difference of serum CD93 levels in control and asthma patients (**A**). Receiver operating characteristic curve of CD93 to predict asthma (**B**). *P < 0.05.

The mean age of enrolled subjects was 38.9 years old. Male occupies 41.7%. Among total enrolled subjects, 28 subjects were finally diagnosed as asthma. Age, sex, smoking history, prevalence of allergic rhinitis, and use of intranasal steroid were not significantly different according to the group (Supplementary Table 1). The serum sCD93 level in ICS-naïve asthma patients (155.3 ± 9.1 μg/mL) was significantly (*P* < 0.001) higher than that in the control group (112.2 ± 3.7 μg/m) (Fig. 6A). After adjusting for age and sex, a higher level of CD93 (≥138.5 μg/mL) was found to predict asthma with moderate sensitivity (71.4%) and specificity (82.4%) (AUC = 0.787, *P* < 0.001) (Fig. 6B).

## Discussion

We demonstrated that allergic asthma is associated with significant changes in CD93 expression and serum levels in both *in vitro* and *in vivo* models. In addition, this study suggests that CD93 has potential role to predict asthma in humans. Clinicians frequently encounter asthma patients who cannot conduct conventional tests for asthma diagnosis or assessment of asthma control for various reasons, including old age, underlying comorbidity, decreased lung function, or dyspnea. Since CD93 can be detected in a soluble form in the serum, it can be easily and non-invasively measured, highlighting sCD93 as a useful surrogate to diagnose or assess asthma in practical clinical settings.

The CD93 mRNA expression and protein levels increased in culture supernatants after HDM stimulation *in vitro*. However, *in vivo* CD93 expression on the cell surface and in bronchial epithelial cells decreased in the HDM-treated group, whereas the serum level increased. This finding is concordant with that of our previous study using an OVA-induced asthma model^15^. We speculate that this difference is due to the release of CD93 from the cell surface to the serum upon allergen stimulation. CD93 is a transmembrane glycoprotein expressed on the surface of various cell types and can be shed into the serum through cooperation with metalloproteinase^7,22^. Thus, HDM stimulation might initially increase the CD93 mRNA expression level, leading to cleavage of CD93 from the cell surface, shedding of CD93 into the serum, and finally, an increase of the CD93 serum level. Although we could not directly confirm this hypothesis, the results from both the *in vitro* and *in vivo* analyses are consistent with this speculation. Nevertheless, the mechanisms by which CD93 sheds from the cell surface to serum warrant further investigation.

Silencing of CD93 expression with siRNA decreased the CD93 level in the culture supernatants to the same extent as observed with dexamethasone treatment. Dexamethasone is a non-specific anti-immunologic and anti-inflammatory agent, that is well-known to reduce the levels of various cytokines^23^. In contrast to the effects of dexamethasone, treatment of CD93 siRNA increased the levels of inflammatory cytokines IL-6 and TSLP in culture supernatants. This suggests that IL-6 and TSLP respond earlier than CD93 to an allergen, so that inhibiting CD93 resulted in a further increase of IL-6 and TSLP to compensate for this deficiency. However, IL-33 was not affected by inhibition of CD93, suggesting that IL-33 is irrelevant at this stage or is active at a later stage in the allergic response than CD93. Although further studies are needed to reveal the specific mechanisms, our results suggest that IL-6 and TSLP may mediate CD93-associated inflammation.

**Figure 7:**
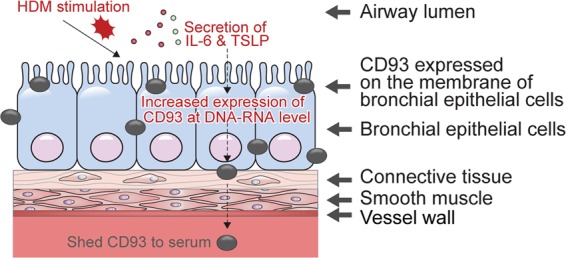
Summarized scheme of the possible mechanism.

Collectively, the cascade of CD93-associated airway inflammation can be summarized as follows based on our results (Fig. 7). HDM stimulation induces cytokine production at the mRNA and protein levels, especially IL-6 and TSLP. This stimulation causes CD93 expressed in bronchial epithelial cells to be shed to the serum in a soluble form. Therefore, CD93 will be detectable in the serum under allergic stimulation. Although further studies are needed to confirm this hypothesis firmly, all the data obtained in this study point in the same direction: CD93 responds to allergic stimulation. Given the insufficient and conflicting data concerning the role of CD93 in allergic stimulation, this study provides a promising foundation for further detailed investigations on the roles of CD93 in allergic asthma.

Moreover, we demonstrated that HDM induces changes in CD93 *in vitro*, and we were able to determine the optimal concentration and timing of HDM (0.1 μg/mL of Der p 1) to induce significant changes of CD93 levels in BEAS-2B cell and U937 cell, along with cytokines and other related genes. These results are similar with previous studies concerning other cytokines^24,25^. Previous study said that HDM can stimulate U937 cell via IL-13Rα2 receptor^16^. Therefore, this model can serve as a useful tool for further detailed *in vitro* analyses to better understand the mechanisms of HDM-induced asthma.

The serum CD93 level showed moderate predictive power (sensitivity, 71.4%; specificity, 82.4%) for asthma diagnosis in human subjects. Previous studies have revealed that the serum CD93 level is significantly higher in patients with exacerbated asthma compared to those with stable asthma^13,14^. In addition, the serum CD93 level was significantly associated with the ICS dose^14^. However, this is the first study to reveal diagnostic power of CD93for asthma diagnosis. In addition, this moderate predictive power is comparable to that of previously studied biomarkers, including serum periostin and fractional exhaled nitric oxide^26,27^. Because of small number of asthma subjects (n = 28), we could not find significant correlation of level of CD93 with lung function or level of eosinophil.

However, there are some limitations of this study that should be mentioned. First, we could not firmly confirm the detailed mechanism by which CD93 affects allergic inflammation. Further studies focusing on IL-6, TSLP, and CD93 are needed to reveal this mechanism. Blocking IL-6 and TSLP might be the next step of this study. Second, treatment of an CD93 inhibitor *in vivo* would be useful to confirm the hypothesis. Third, more controlled human studies with abundant number of subjects will be needed to validate the clinical utility since we were only able to conduct a retrospective post-hoc analysis with limited number of subjects. Last, endotoxin which is naturally included in HDM might affect the response of bronchial epithelial and mouse experiment.

The incidence of asthma is increasing worldwide, but there are still limited serologic biomarkers available^28,29^. We showed that CD93 is significantly associated with allergic inflammation using both *in vitro* and *in vivo* models, and the predictive power of CD93 for asthma diagnosis was modest. Importantly, the serum soluble CD93 level can be measured easily, which would be a benefit for patients and clinicians to detect asthma.

## Supplementary information

Supplementary table 1.
