# Supplementary material for: Soluble CD93 in allergic asthma
Source: Sci Rep. 2020 Jan 15;10:323. doi: 10.1038/s41598-019-57176-2 (PMC6962376; doi:10.1038/s41598-019-57176-2)
Supplement: Supplementary file 1 — Supplementary table 1. [file 41598_2019_57176_MOESM1_ESM.docx]

Manuscript title: Soluble CD93 in allergic asthma

Author list: Hye Jung Park MD, PhD^1^, Eun-Yi Oh MS^2^, Hee-Jae Han MS^2^, Kyung Hee Park MD, PhD^2,3^, Kyoung-Yong Jeong PhD^2^, Jung-Won Park MD, PhD^2,3^, Jae-Hyun Lee MD, PhD^2,3^*

^1^Department of Internal Medicine, Gangnam Severance Hospital, ^2^Institute of Allergy, ^3^Division of Allergy and Immunology, Department of Internal Medicine, Yonsei University College of Medicine, Seoul, Republic of Korea

Supplementary table 1. Baseline characteristics of enrolled subjects

|  | Healthy control  (n=68) | Asthma patients  (n=28) | *P*-value |
| --- | --- | --- | --- |
| Age (yr) | 38.2±1.8 | 40.7±3.1 | 0.470 |
| Sex (male, %) | 29 (42.6%) | 11 (39.3%) | 0.761 |
| Current smoker, n (%) | 1 (1.5%) | 2 (7.4%) | 0.136 |
| Allergic rhinitis, n (%) | 53 (77.9%) | 20 (71.4%) | 0.497 |
| Intranasal steroid use, n (%) | 34 (50.0%) | 9 (32.1%) | 0.110 |
| Level of sCD93 (μg/mL) | 112.2±30.8 | 155.3±9.1 | <0.001 |
